# Supplementary material for: Placebos without deception reduce self-report and neural measures of emotional distress
Source: Nat Commun. 2020 Jul 29;11:3785. doi: 10.1038/s41467-020-17654-y (PMC7391658; doi:10.1038/s41467-020-17654-y)
Supplement: Supplementary file 1 — Supplementary Information [file 41467_2020_17654_MOESM1_ESM.pdf]

Supplementary Information

Placebos without deception reduce self-report and neural measures of emotional distress

Guevarra et al.

Supplementary Notes: 3
Supplementary Methods: 4
Supplementary Tables: 12
Supplementary Figures: 3

Supplementary Note 1: Open-label placebos versus non-deceptive placebos

Placebos without deception have several names in the literature from open-label placebos, honest placebos, and non-deceptive placebos[1]. The term open-label placebo originated from studies that test placebos without deception on different medical conditions and that use inert pharmacological objects as their placebo, such as pills[2]. Therefore, the term open-label placebo makes perfect sense within the clinical context, and when the placebo object is pharmacological in nature. However, placebo objects can be non-pharmacological such as bracelets, rings, and many other objects. Consequently, we prefer the term non-deceptive placebo because it is more inclusive of different types of placebo objects and is more broadly applicable outside the clinical context.

Supplementary Table 1: Summary results of studies on non-deceptive placebos

Domain	Types of Measures	Subjective Outcome(s): Compared to baseline	Objective Outcome(s): Compared to baseline	Subjective Outcome(s): Compared to control group	Objective Outcome(s): Compared to control group
Neurotic symptoms[3]	• Self-report from patients and doctors	Yes	N/A	N/A	N/A
ADHD[4]	• Self-report from parents and teachers[a]	Mixed	N/A	N/A	N/A
ADHD[5]	• Self-report from parents and teachers[b]<br>• Attention performance	Mixed	Yes[c]	Mixed	No
Irritable bowel syndrome[2]	• Self-report	N/A	N/A	Yes	N/A
Major depressive disorder[6]	• Self-report and clinician ratings	Yes	N/A	No	N/A
Episodic migraine attacks[7]	• Self-report	Yes	N/A	N/A	N/A
Chronic lower back pain[8]	• Self-report	Yes	N/A	Yes	N/A
Allergic rhinitis[9]	• Self-report	Yes	N/A	No	N/A
Heat pain[10]	• Self-report	No	N/A	Mixed[d]	N/A
Heat pain[11]	• Self-report<br>• Pain tolerance	N/A	N/A	Mixed[e]	No
Skin reaction from histamine prick test[12]	• Self-report<br>• Physical skin condition	N/A	N/A	No	No

[a] Non-deceptive placebo effect is interpreted as no change in ADHD symptoms from 100% dose condition versus 50% dose + placebo condition. Non-deceptive placebo effect was found for parent ratings. Teacher ratings were mixed since there was no difference in ADHD symptoms for 100% dose condition, 50% dose + placebo condition, and 50% dose condition.
[b] Non-deceptive placebo effect is interpreted as no change in ADHD symptoms from 100% dose group versus dose reduction + placebo group. Non-deceptive placebo effects were found for parent's report but not teacher's report.
[c] The study suggests that no change on attentional performance from baseline in the reduced dose plus non-deceptive placebo group is an objective effect; however, since the reduced dose without a placebo group also showed no change on attentional performance, it is difficult to interpret if this is an instance of an objective non-deceptive placebo effect. Moreover, a full dose group trended to worst attentional performance (contrary to prediction) making it even more difficult to interpret any objective effects.
[d] For the non-deceptive placebo group, there were no difference from baseline to the testing phase. For the control group, however, there was an increased in self-reported pain from baseline to the testing phase. The authors interpreted this as non-deceptive placebos preventing a sensitization effect.
[e] There were four comparison groups: a no-treatment control, a non-deceptive placebo group with no rationale, a non-deceptive placebo group with a rationale, and a deceptive placebo group. There was a significant difference between the non-deceptive placebo group with a rationale compared to the non-deceptive placebo group without a rationale. There was no difference between the no-treatment control group and non-deceptive placebo group with a rationale.

Allergic rhinitis[13]	• Self-report symptoms and quality of life	Yes	N/A	N/A	N/A
Cancer-related fatigue[14]	• Self-Report	Yes	N/A	Yes	N/A
Cancer-related fatigue[15]	• Self-Report	Yes	N/A	Yes	N/A
Wound healing[16]	• Wound healing rate	N/A	N/A	N/A	No
Emotional distress, physical symptoms & sleep quality[17]	• Self-report[f]	N/A	N/A	Yes	N/A
Skin reaction from histamine prick test[18]	• Physical skin condition[g]	N/A	N/A	N/A	Mixed[h]
Nausea[19]	• Self-report	N/A	N/A	No	N/A
Test anxiety[20]	• Self-report	Yes	N/A	Yes	N/A
Physical performance[21]	• Self-report<br>• Physical performance	Yes	No	N/A	N/A
Cycling performance[22]	• Self-report<br>• Physical performance<br>• Physiological measures	No	Mixed[i]	N/A	N/A
Acute pain[23]	• Self-report	Yes	N/A	N/A	N/A
Allergic rhinitis[24]	• Self-report<br>• Physiological measures	N/A	N/A	No	No
Chronic back-pain[25]	• Self-report<br>• Physical mobility	N/A	N/A	Yes[j]	No
Physical performance[26]	• Physical performance<br>• Physiological measure	N/A	No	N/A	N/A
Heat pain[27]	• Self-report<br>• Pain tolerance	N/A	N/A	No	No

Note: The table provides a summary of results on non-deceptive placebo studies. Columns three through six provide results on subjective and objective outcomes and reference of comparisons. "Yes" indicates significant beneficial effects. "No" indicates null findings. "N/A" indicates no comparison was possible, or no results were provided for that comparison. "Mixed" refers to unclear interpretations of the results. We give more information on mixed results with footnotes.

[f] There were two placebo groups that differed in number of pills taken per day: 1 placebo per day or four placebos per day. Both were equally effective and did not differ from each other.

[g] The non-deceptive placebo manipulation was ineffective in convincing people that non-deceptive placebos could work, making interpretation of the results inconclusive or ambiguous.

[h] No main effect was detected; however, when the sample was split between those with low placebo belief versus high placebo belief, when only looking at the high placebo belief group, the non-deceptive placebo with a rationale group had a smaller wheal size compared to the control group.

[i] Yes, on time to completion. No on biological measures such as heart rate and blood lactate.

[j] It did not, however, affect secondary outcomes such as anxiety and stress.

Supplementary Methods 1: Reading manipulation for both experiments

To maximize our persuasion attempt[28], our non-deceptive placebo manipulation consisted of an article reading accompanied by a verbal suggestion from the experimenter. Borrowing from Kaptchuk and colleagues' seminal study on placebos without deception[2], we attempted to convey the following points about placebos: 1) placebos are powerful and work in many domains, 2) placebos can affect behavior and biology, and 3) placebos have been shown to work even when people know they are taking a placebo (i.e., without deception). The information from our article manipulation differed slightly from Kaptchuk and colleagues' in that we did not highlight the classical conditioning mechanism of the placebo effect and that we provided two studies showing that placebos without deception can work[2, 6]. For ease of reading, the article manipulation was broken down into three main parts divided into 13 online pages. The articles were matched for narrative structure, negatively valenced words (control = 62, non-deceptive placebo = 58), and length (control = 1287 words, non-deceptive placebo = 1270 words). We detail the contents of each part of the article manipulation and the verbal suggestion from the experimenter below. We also provide a summary of the article manipulation and verbal suggestion instructions for the control condition.

Control Article	Non-Deceptive Placebo Article
You will now read information on the neurological processes of pain. Each page will start with a brief two or three sentence summaries with an image of an article. Some of the articles will have highlighted portions. Some pages will also contain figures or graphs to better illustrate our point. We want you to especially pay attention to these. You may need to scroll down for some of these pages. At the end of each page, you will be able to see a citation where we obtained our information, and we can provide you a complete list of references at the end of the study. Once you are finished, please continue to the next page by clicking the arrow at the bottom right of the screen.<br><br>Each web page has a minimum amount of time before the next button shows up to ensure that everyone is reading at a similar pace. After that time expires you will have the option to proceed to the next page, but you may stay on the page for as long as you need to understand the material. The survey does not allow you to click back, so make sure that you are ready to proceed before you click next.	You will now read information on placebos and the placebo effect. Each page will start with a brief two or three sentence summaries with an image of an article. Some of the articles will have highlighted portions. Some pages will also contain figures or graphs to better illustrate our point. We want you to especially pay attention to these. You may need to scroll down for some of these pages. At the end of each page, you will be able to see citations from which we obtained our information, and we can provide you a complete list of references at the end of the study. Once you are finished, please continue to the next page by clicking the arrow at the bottom right of the screen.<br><br>Each web page has a minimum amount of time before the next button shows up to ensure that everyone is reading at a similar pace. After that time expires you will have the option to proceed to the next page, but you may stay on the page for as long as you need to understand the material. The survey does not allow you to click back, so make sure that you are ready to proceed before you click next.
We would like to provide you with up-to-date information on the neurological processes of pain and how to treat it**.** The following passage contains screen shots of the scientific articles where we obtained our information. At the end of the survey, you will be asked about what you read to determine how well you remember the information.<br><br>The neurology of pain refers to the physiological processes within the brain that result in the sensation of physical pain. For example, if you were to stub your toe, the nerves within the skin would send signals through the spinal cord to the thalamus and varying structures of the brain. These other	We would like to provide you with up-to-date information on placebos and the placebo effect**.** The following passage contains screen shots of the scientific articles where we obtained our information. At the end of the survey, you will be asked about what you read to determine how well you remember the information.<br><br>A "placebo" refers to any inactive substance or procedure that has beneficial effects because people think it will make them feel better. For example, if you take a nasal spray (inactive substance) to reduce pain and you believe that it is a pain medication, then your pain will diminish. Your

brain structures would create further electrical signals to identify and translate the pain sensation to varying parts of your body.<br><br>It is important to understand how pain works and how best to treat it. We will provide you with details of some of these processes and treatment in the next few pages.	positive beliefs in the effectiveness of a treatment is one of the key ingredients in getting a placebo to work.<br><br>The placebo has been shown to work effectively on a host of conditions, symptoms, and disorders. We will detail some of these in the next few pages.
For example, in an article published in Science*,* one of the leading journals across disciplines, researchers from University of Oxford show that expectation and experience of pain show activation in distinct brain regions. Moreover, the study demonstrates that anticipation of pain actually causes mood changes that can enhance the experience of pain.<br><br>(Contains a highlighted portion of an article)	For example, in an article published in Science, one of the leading journals across disciplines, researchers from the University of Michigan, the University of Wisconsin, Princeton, and Harvard show that placebos reduce subjective experiences of pain. Moreover, the study demonstrates that people show less activity in brain regions associated with pain after taking a placebo.<br><br>(Contains a highlighted portion of an article)
In another article, researchers show that in patients with neuropathic pain, definite associated regions of the brain can be identified. For example, brain regions associated with motor, sensory, and automatic functioning are all implicated in the process of pain.<br><br>(Contains a highlighted portion of an article)	In another article, researchers show that placebos are just as effective as antidepressants in reducing symptoms of depression. They illustrate that placebo treatments reduce symptoms of depression for those who experience low to mild depression.<br><br>(Contains a highlighted portion of an article)
Additionally, a team of researchers has also shown that other types of brain cells called glia are important for lessening chronic pain. They find that manipulating these glia cells via drugs can potentially help alleviate chronic pain sensations.<br><br>(Contains a highlighted portion of an article)	Additionally, a team of neuroscientists has shown that a placebo treatment is effective in reducing negative emotional experiences. People that expected to receive an anxiolytic drug (a substance that reduces negative emotions) experienced less negative emotions and showed less brain activity in regions associated with emotional processing.<br><br>(Contains a highlighted portion of an article)
It is important to emphasize the relationship between pain and pleasure found in the brain. Pain and pleasure are often thought of as opposite processes, but research has shown that similar brain regions are responsible for producing both sensations. In the previous pages, we mentioned that motor, sensory, and autonomic systems were implicated in pain processing. Understanding this relationship is important because it can help identify treatments for pain. We highlight some of these treatment options in the next few pages.<br><br>For example, in the article below, researchers demonstrate that the opioid (type of chemical in the brain that is similar to morphine) and dopamine (another chemical associated with reward and motivation) systems both play an important role in pain and pleasure sensations. These researchers highlight many of these systems in order to advance our understanding of treatments for alleviating unnecessary pain.	It is important to emphasize that a placebo treatment affects more than how people feel on a subjective level. They lead to changes in behavior and the brain. In the previous pages, we mentioned a few of these studies where researchers show that placebos work on brain regions associated with pain and negative emotions. We highlight some more of these findings in the next few pages.<br><br>For example, in the article below, researchers tested placebos on people with Parkinson's disease, a degenerative disorder of the brain characterized by movement related symptoms such as shaking and difficulty with movement. These researchers demonstrate that people with Parkinson's disease who received a placebo showed less abnormal muscle movements during a 6-month study.

(Contains a highlighted portion of an article)	(Contains a highlighted portion of an article)
One example of these brain-related treatments of pain is using transcranial direct current stimulation (a form of neurostimulation in which constant, low current is delivered directly to the brain area of interest with small electrodes). A team of neuroscientists showed that symptoms of fibromyalgia (a syndrome characterized by muscle pain) can be reduced by using non-invasive brain stimulation.<br><br>The figure below shows that people who receive transcranial direct stimulation show decreases in symptoms as assessed by a clinician.<br><br>(Contains a highlighted portion of an article)	Another example of the power of placebos is a study using brain imaging techniques. A team of neuroscientists showed that people who received a placebo before receiving a painful stimulation showed a decrease in parts of the brain that are active during painful sensations. These areas include the secondary somatosensory cortex, midbrain, anterior insula, and anterior cingulate cortex.<br><br>The figure below shows some of these regions as well as the decrease in pain responses illustrated by the blue line around 40 seconds. The red arrow points to the brain activity during a natural pain response. The blue arrow points to the brain activity during a placebo response. As you can see, the brain activity after a placebo is substantially reduced.<br><br>(Contains a highlighted portion of an article)
In the past, pain and chronic pain disorders have been treated with biological methods such as drugs, transcranial direct current stimulation, and other biologically based techniques. Because of the strong emphasis on the physiological and neurological basis of pain, most research on interventions has been on these physiologically and neurologically based methods.<br><br>However, new research has started looking at the strong link between the mind and the brain in relationship to pain. For example, recent research has suggested that mind-based interventions such as mindfulness practice and cognitive-behavioral therapy may prove just as effective in relieving the severity of symptoms for those who suffer from chronic pain.<br><br>In the next few pages, we will provide you with information on research that studies the effectiveness of these mind-based programs: interventions that directly target people's psychological states.	In most of these placebo studies, people are deceived into believing that they are going to take a powerful treatment, and this belief results in a placebo response. This finding has historically led physicians and scientists to believe that it was necessary to deceive people in order for placebos to work.<br><br>However, new research has begun to challenge this assumption. A number of recent studies demonstrate that deception is NOT necessary as long as people still believe that a placebo can work even if they know it is a placebo.<br><br>In the next few pages, we will provide you with information on research that studies the effectiveness of open-label or non-deceptive placebos: placebos where researchers directly tell the participants they were taking a placebo instead of lying to them.
In one study, a team of scientists and medical doctors from various universities conducted a mindfulness-based stress reduction intervention for people with chronic pain. Chronic pain includes arthritis, headaches, and back/neck pain.<br><br>In this study, individuals suffering from chronic pain participated in an 8-week mindfulness-based pain reduction program where they learned a variety of meditation techniques: body scan, yoga, and awareness of emotions. Individuals who participated in this program showed a decrease in their bodily pain and also in the anxiety they felt regarding that pain 8 weeks after the program.	In one study, a team of scientists and medical doctors from various universities conducted an open-label placebo study (also called non-deceptive placebos) where they directly told participants they were receiving a placebo) on patients with Irritable Bowel Syndrome. Irritable Bowel Syndrome is a gastrointestinal disorder characterized by abdominal pain, discomfort, bloating, and dysregulated bowel habits.<br><br>In this study, patients were directly told that they were receiving a placebo pill, but that it would still be effective if they believed it would be. Patients who received the placebo compared to those who did not receive anything showed a greater reduction in Irritable Bowel Syndrome

The figures below show bar graphs comparing people's pain severity and anxiety severity at Day 1 and after the 8-week program at Day 56.<br><br>Figure 1 shows that after the 8-week mindfulness-based stress reduction program, people's bodily pain severity is reduced at Day 56.<br><br>Figure 2 shows that after the 8-week mindfulness-based stress reduction program, people's anxiety severity is reduced at Day 56.<br><br>(Contains recreated figures of the described effect)	symptoms and a greater increase in quality of life after three weeks.<br><br>The figures below show bar graphs comparing the No-Treatment group versus the Open-Label Placebo group on symptom severity change and quality of life change.<br><br>Figure 1 shows that after 3 weeks, patients in the No-Treatment group slightly improve in their symptom severity. That is to say, they experience less symptoms compared to when they started; however, as you can see, those in the Open-Label Placebo group reported greater symptom severity change compared to those in the No-Treatment group.<br><br>Figure 2 shows a similar pattern for Quality of Life Change. After 3 weeks, patients in the No-Treatment group showed a slight improvement in their quality of life; however, as you can see, those in the Open-Label Placebo group showed a greater improvement in quality of life compared to the No-Treatment group.<br><br>(Contains recreated figures of the described effect)
In another study, a team of scientists and medical doctors from various universities conducted a survey of children with recurring abdominal pain. In this study, patients and their parents completed three sessions of cognitive behavioral therapy. After completing their sessions, children reported much less pain and parents observed less pain-related behavior. Moreover, they showed less pain after a 6-month and 12-month follow up.<br><br>Figure 1 shows children's self-report ratings of pain before any treatment takes place. As you can see, after the three sessions of cognitive behavioral therapy, children's pain severity substantially drops at Post-Treatment and even after a 6 and 12 month follow up.<br><br>Figure 2 shows a similar pattern for the parent's observation of pain behavior. You can see the parent's observation of pain behavior before any treatment takes place. After three sessions of cognitive behavioral therapy, parent's observation of pain behavior substantially drops at Post-Treatment and even after a 6 and 12 month follow up.<br><br>(Contains recreated figures of the described effect)	In another study, a team of scientists and medical doctors from various universities conducted a non-deceptive placebo study on people with Major Depressive Disorder. In this study, patients were also directly told they would be receiving a placebo treatment to help treat Major Depressive Disorder symptoms. After 28 days of taking placebo pills, patients showed a reduction in Major Depressive Disorder symptoms measured by both patient self-report and a clinician's observational report.<br><br>Figure 1 shows a patient's self-report ratings of depression severity at Day 1. As you can see, after taking placebo pills openly (as in having knowledge of taking placebo pills), they show a substantial reduction in depression symptoms at Day 28.<br><br>Figure 2 shows a similar pattern. At Day 1 a clinician makes an observation regarding the severity of the patients' depression. As you can see, after openly taking placebo pills for 4 weeks, there was a substantial decrease in clinician-assessed severity in depression symptoms.<br><br>(Contains recreated figures of the described effect)
These two studies provide evidence that mind-based programs can be effective in reducing pain and chronic pain not only for adults but also for children. Thus, these mind-based programs may be a good alternative or supplement for existing biologically based interventions in reducing pain.	These two studies provide evidence that non-deceptive placebos can be effective as long as people believe that taking the placebo will make them feel better. Thus, if you believe that a non-deceptive placebo will make you feel better, then it will.

To summarize, you were provided with information on the neurological processes of pain and how to treat it. We now want to emphasize a few key points from the reading.<br><br>• Pain refers to a complex physiological process within the central nervous system that result in the sensation of physical pain.<br>• Pain and pleasure share many nervous system pathways and structures.<br>• Pain has a strong biological substrate which lead researchers to identify biologically based methods for treating pain such as drugs and brain stimulation techniques.<br>• Pain can be treated using non-biological interventions such as mindfulness training and cognitive behavioral therapy.<br><br>You are now finished with this part of the study. Please click next to proceed with the next part of the study.	To summarize, you were provided with information about placebos and the placebo effect. We now want to emphasize a few key points from the reading.<br><br>• Placebos are inactive substances or procedures that make people feel better because they believe it will.<br>• Placebos affect a variety of conditions, disorders, and symptoms including pain, depression, anxiety, and negative emotions.<br>• Placebos affect not only how you subjectively feel but also your behavior and physiology.<br>• Placebos can work even if you know you are taking a placebo as long as you believe that the placebo will be effective.<br><br>You are now finished with this part of the study. Please click next to proceed with the next part of the study.

Part 1 (pages 1–4). Part 1 was intended to show that placebos are robust and work across many domains, including pain, depression, and negative distress. Page 1 provided an overview of the type of information that they will be reading. It explained that the content of the following pages consisted of a summary paragraph and a picture of either a screenshot of the original paper or a figure or graph. We believed this combination was a balanced blend of providing easily understood information with some scientific support.

Page 2 provided information on a study showing that placebos reduced subjective experiences of pain along with an abstract of the research article[29]. Page 3 provided information on a meta-analysis showing that there are no differences in the efficacy of antidepressants and placebos for treating low and moderate levels of depression severity; this suggests that placebos can be just as effective as antidepressants for some levels of depression[30]. Page 4 summarized a study showing that placebos were able to reduce negative emotional experiences[31].

Part 2 (pages 5–6). Part 2 was intended to demonstrate that placebos worked on more than subjective experience and can affect objective measures such as behavior and the brain. Page 5 provided information on the effects of placebos on objective changes in motor function for people with Parkinson's disease[32]. Page 6 highlighted the dynamic effects of placebos on two brain regions associated with the pain response[29].

Part 3 (pages 7–10). Part 3 was designed to provide support for the idea that placebos can work without deception. Page 7 first introduced the idea that deception was imbedded in most placebo studies and was the number one way to induce a placebo effect. It then introduces the notion that deception may not be necessary. Page 8 summarized Kaptchuk and colleagues' seminal paper on the impact of placebos without deception on symptoms of Irritable Bowel Syndrome; we also reproduced two of their bar graphs showing the beneficial effects of placebos without deception on symptom severity and quality of life[2]. Page 9 provided information on the effects of placebos without deception on major depressive disorder; we reproduced their within-subjects findings depicting a decrease in self-reported and clinician-reported depression symptoms from day 1 to day 14[6]. Page 10 reiterated that the "*two studies provide evidence that non-deceptive placebo are as effective as deceptive placebos as long as people believe the*

placebo will make them feel better." We further positively framed that if a person believed a non-deceptive placebo would make them feel better, then it will.

Verbal suggestion from the experimenter. After the article reading, the experimenter prepared the participant for the placebo nasal spray administration. The experimenter said:

> *...you will be given a placebo nasal spray to reduce your negative emotional reactions. Again, this is a placebo, which means it does not contain any active ingredients, and it is completely harmless. But as you have read from the article, if you believe that the nasal spray will reduce your negative emotional reactions, then it actually will.*

The experimenter then administered the placebo nasal spray, and the participant went through an image viewing task.

Control article manipulation and verbal suggestion. For the control condition, we created an article on the neurological processes of pain and how to treat it. We picked this topic because it readily provided us with content that we could match in terms of neurological findings. We tried our best to control for the amount and type of content. In terms of the verbal suggestion, participants were told that the nasal spray was designed to get better physiological readings.

Supplementary Methods 2: Additional manipulation information for Experiment 2

Article manipulation. The placebo article manipulation for Experiment 2 was nearly identical to the one in Experiment 1. The main difference in Experiment 2 was that we asked participants to recall and write about what they learned and remember from the readings as best as they could. We framed this as another memory task. We reasoned that our article manipulation would be more persuasive if participants actively engaged with the placebo information and bring it to the forefront of their minds[28].

Verbal suggestion from the experimenter. We also expanded on the verbal suggestion from the experimenter. Before administering the placebo nasal spray, the experimenter says:

> *From what you have read, you know that placebos are inert substances or procedures that make people feel better mostly because they believe it will. You also know that placebos are powerful and can help reduce pain, depression, anxiety, and negative emotions. On top of that, you've read that placebos affect more than how you feel, they actually can change your behavior, physiology, and even brain activity. And new research has also shown that placebos can work even if you know you are taking one since the key ingredient is the positive belief that it can help and that it works.*

After the administration, the experimenter states:

> *I just want to remind you that I just administered a placebo nasal spray that contains no active ingredients, but if you believe it will reduce your negative emotional reactions to these images, then it will. The placebo really works because of your positive beliefs and expectations.*

Control article manipulation and verbal suggestion. The control article was also nearly identical to Experiment 1. Participants in the control group also had to write about what they remembered and learned from the readings. In terms of the verbal instructions from the experimenter, participants were told that the nasal spray was designed to get better physiological readings. The experimenter explained:

> *Your sinuses consist of passageways that surround the nasal cavity. Two of these passageways are located above and under your eyes. One is located between the eyes. The last one is located behind them. The nasal cavity is a large space above and behind the nose in the middle of the face. These spaces are naturally filled with a thick membrane that gets recycled throughout the day. Unfortunately, this thick membrane often interferes with the electrical signals produced by your brain. So, to help us get better signals, we administer a saline nasal spray to help clear some of this space.*

Supplementary Table 2: Correlations between beliefs and expectations with self-report outcomes for Experiment 1

Variables	M	SD	1	2	3	4
Beliefs	3.80	2.01	—			
Expectations	1.74	2.25	.07	—		
Self-report (negative)	6.89	1.45	-.13	-.19	—	
Self-report (neutral)	1.86	1.03	-.11	.04	.09	—

Note. A total of n = 62 participants was used to calculate means, standard deviations, and all bivariate Pearson correlations (two-tailed). $^{*}p < 0.05$.

We did not find significant relationships between general beliefs in the efficacy of non-deceptive placebos and self-reported emotional distress for negative images (p = .317) and neutral images (p = .382). Moreover, we did not find significant relationships between expectations of efficacy regarding the nasal spray and self-reported emotional distress for negative images (p = .141) and neutral images (p = .753). We elaborate on why this may be the case in the Discussion section of the main manuscript.

Supplementary Table 3: Detailed comparison statistics for sustained LPP for different time windows

	Control	Non-deceptive placebo			
**Time Window**	***M (SEM)***	***M (SEM)***	***t***	***p***	***d***
1000–2000 ms	2.59 (.39)	1.79 (.39)	1.48	.141	.21
2000–3000 ms	2.84 (.51)	1.09 (.51)	2.43	.016	.35
3000–4000 ms	2.96 (.54)	.54 (.54)	3.15	.002	.45
4000–5000 ms	2.93 (.57)	.36 (.57)	3.19	.002	.45
5000–6000 ms	3.02 (.59)	.50 (.59)	3.01	.003	.43

Note. This table represents detailed statistics for multiple comparisons for a significant Condition by Time interaction (p = .017) with two samples combined (total participants, n = 198; control group, n = 99; non-deceptive placebo group, n = 99). All follow-up independent pairwise comparisons were two-tailed with unadjusted p values. The comparison was control minus non-deceptive placebo. Positive t and d indicate beneficial effects of non-deceptive placebos.

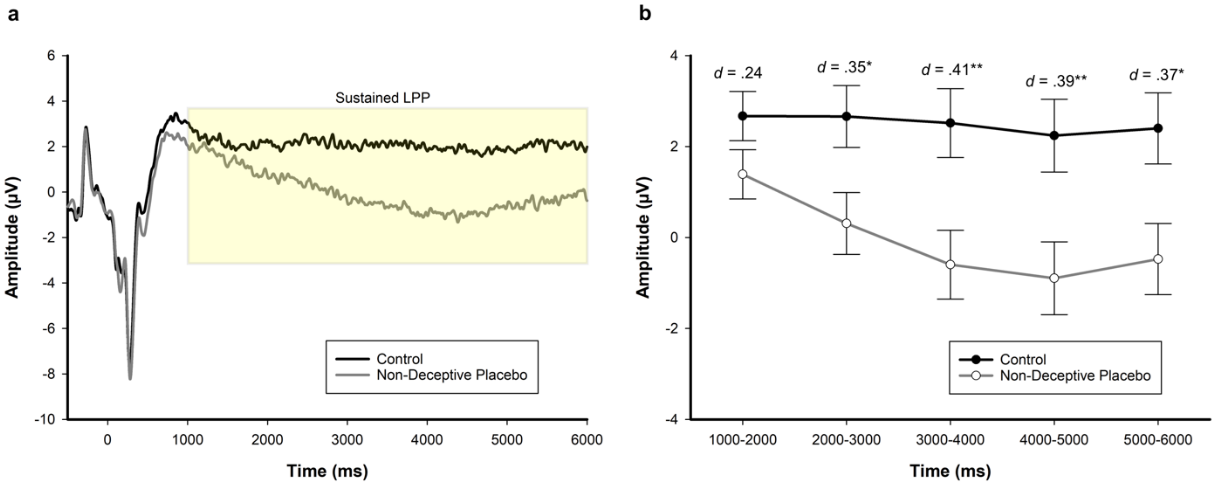

Supplementary Figure 1: Corroboratory sustained LPP analysis at CPz (*n* = 198 participants). Combining two samples, we performed a corroboratory analysis of the sustained LPP at CPz, where the LPP is typically maximal and analyzed (total participants, *n* = 198; control group, *n* = 99; non-deceptive placebo group, *n* = 99). **a** Picture-locked ERP waveforms depict estimated mean amplitude (µV) waveforms at the CPz electrode site for the control group (*n* = 99) and non-deceptive placebo group (*n* = 99) with amplitudes from neutral and negative images collapsed for each group. A higher number indicates a larger amplitude. Picture onset occurs at 0 ms. The yellow shaded area represents the time window for analysis of the sustained LPP at CPz. **b** We conducted a 2 (Condition: control and non-deceptive placebo) X 2 (Sample: sample 1 and sample 2) X 2 (Picture Type: neutral and negative) X 5 (Time: 1000–2000 ms, 2000–3000 ms, 3000–4000 ms, 4000–5000 ms, and 5000–6000 ms) at CPz followed-up by independent pairwise comparisons for the Condition by Time interaction. All tests were two-tailed, and follow-up tests were not adjusted for multiple comparisons. Similar to the cluster analysis, we found a main effect of Condition on sustained LPP amplitude at CPz, $F(1, 194) = 7.56$, $p = .007$, $\eta_p^2 = .037$. This main effect was qualified by a marginally significant interaction between Condition and Time, $F(1.55, 300.60) = 2.86$, $p = .072$, $\eta_p^2 = .015$. To parallel the follow-up analysis from the cluster analysis, we conducted follow-up independent pairwise comparisons. Line plots depict estimated marginal mean amplitude (µV) per Condition (control group, *n* = 99, and non-deceptive placebo group, *n* = 99) with activity from each Picture Type (neutral and negative) collapsed across Time (1000–2000 ms, 2000–3000 ms, 3000–4000 ms, 4000–5000 ms, 5000–6000 ms). Error bars represent ± 1 SEM. Consistent with the cluster finding, follow-up independent pairwise comparisons showed that the non-deceptive placebo group exhibited a gradual decrease in sustained LPP amplitude compared to the control group across the five time points, 1000–2000 ms ($p = .095$), 2000–3000 ms ($p = .015$), 3000–4000 ms ($p = .004$), 4000–5000 ms ($p = .006$), and 5000–6000 ms ($p = .010$). See Supplementary Table 4 for detailed comparison statistics. The comparison was control minus non-deceptive placebo. Positive *d* indicates the beneficial effects of non-deceptive placebos. No asterisk = not significant, $^{*}p < 0.05$, $^{**}p < 0.01$.

Supplementary Table 4: Detailed comparison statistics for sustained LPP at CPz for different time windows

	Control	Non-deceptive placebo			
**Time Window**	***M (SEM)***	***M (SEM)***	***t***	***p***	***d***
1000–2000 ms	2.67 (.54)	1.39 (.54)	1.68	.095	.24
2000–3000 ms	2.66 (.68)	.31 (.68)	2.45	.015	.35
3000–4000 ms	2.52 (.76)	-.60 (.76)	2.91	.004	.41
4000–5000 ms	2.24 (.80)	-.90 (.80)	2.77	.006	.39
5000–6000 ms	2.40 (.78)	-.47 (.78)	2.59	.010	.37

Note. This table represents detailed statistics for multiple comparisons for a marginally significant Condition by Time interaction at CPz (p = .072) with two samples combined (total participants, n = 198; control group, n = 99; non-deceptive placebo group, n = 99). All follow-up independent pairwise comparisons were two-tailed with unadjusted p values. The comparison was control minus non-deceptive placebo. Positive t and d indicate beneficial effects of non-deceptive placebos.

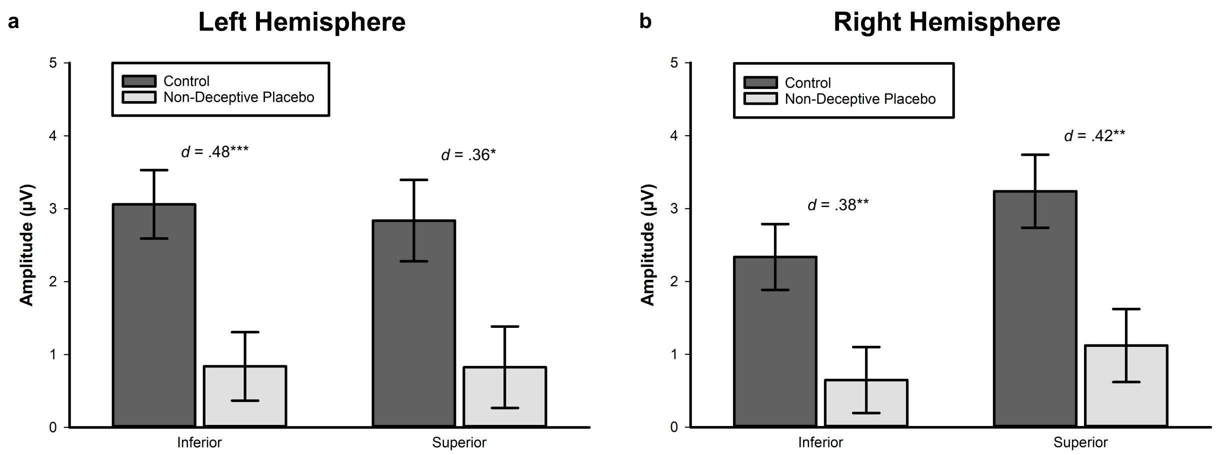

Supplementary Figure 2: Additional sustained LPP interactions (Total *n* = 198 participants). Combining two samples, a mixed-factorial ANOVA (Condition by Sample by Picture Type by Time by Hemisphere by Anterior/Posterior by Inferior/Superior) was conducted followed-up by independent pairwise comparisons for relevant interactions (total participants, $n = 198$; control group, $n = 99$; non-deceptive placebo group, $n = 99$). We found a significant Condition by Hemisphere by Inferior/Superior three-way interaction $F(1, 194) = 4.21$, $p = .042$, $\eta_p^2 = .021$. We probed this interaction with follow-up pairwise comparisons divided by Hemisphere and then by Inferior/Superior sites. All tests were two-tailed, and follow-up tests were unadjusted for multiple comparisons. **a** Bars represent estimated marginal mean amplitude (μV) for the control group ($n = 99$) and non-deceptive placebo group ($n = 99$) with activity from neutral and negative pictures collapsed for the left hemisphere broken down by inferior and superior sites. Error bars represent ± 1 SEM. The non-deceptive placebo group showed less sustained LPP activity compared to the control group at both inferior ($p < .001$) and superior sites ($p = .012$). **b** Bars represent estimated marginal mean amplitude for the control group ($n = 99$) and non-deceptive placebo group ($n = 99$) with activity from neutral and negative pictures collapsed for the right hemisphere broken down by inferior and superior sites. We found a similar pattern with the non-deceptive placebo group showing less sustained LPP activity compared to the control group at both inferior ($p = .009$) and superior sites ($p = .003$). The comparison was control minus non-deceptive placebo. Positive *t* and *d* indicate beneficial effects of non-deceptive placebos. $^{*}p < 0.05$, $^{**}p < 0.01$, and $^{***}p < .001$.

Supplementary Note 2: Additional sustained LPP interactions with Sample and Condition

We also found a significant Condition by Sample by Time interaction, $F(1.62, 314.94) = 5.05$, $p = .011$, $\eta_p^2 = .025$. Breaking down the interaction by Sample, the analysis suggests that the Condition by Time interaction is primarily found in Sample 1 ($n = 102$), $F(1.63, 162.87) = 9.93$, $p < .001$, $\eta_p^2 = .09$, but not Sample 2 ($n = 96$), $F(1.60, 150.51) = .84$, $p = .41$, $\eta_p^2 = .009$. It appears that the Condition by Time interaction we detect with both samples is primarily being driven by Sample 1.

We also found a significant Condition by Sample by Time by Hemisphere interaction, $F(1.43, 276.70) = 4.32$, $p = .025$, $\eta_p^2 = .022$. However, probing this interaction by Sample showed that each sample did not produce a significant Condition by Time by Hemisphere interaction (p values > .05). There were no other significant interactions with Sample and Condition (p values > .05).

Supplementary Table 5: Correlations between beliefs and expectations with sustained LPP (1000–6000 ms)

Variables	M	SD	1	2	3	4	5	6	7	8
Beliefs	5.96	3.07	—							
Expectations	1.43	.62	.35***	—						
Cluster collapsed	1.86	4.80	-.09	-.02	—					
Cluster neutral	.48	5.87	-.06	-.003	.83***	—				
Cluster negative	3.23	5.76	-.08	-.02	.82***	.36***	—			
CPz collapsed	1.23	6.61	-.11	-.14†	.82***	.69***	.66***	—		
CPz neutral	-.67	7.82	-.06	-.11	.73***	.85***	.35***	.86***	—	
CPz negative	3.13	7.58	-.13†	-.13†	.68***	.33***	.79***	.85***	.48***	—

Note. Combining two samples, a total of $n = 198$ participants was used to calculate means, standard deviations, and all bivariate Pearson correlations (two-tailed). $^{\dagger}p < 0.10$, $^{*}p < 0.05$, $^{**}p < 0.01$, and $^{***}p < .001$.

We did not find a significant relationship between general beliefs in the efficacy of non-deceptive placebos and mean sustained LPP activity when activity for neutral and negative pictures was collapsed ($p = .231$), neural pictures only ($p = .416$), negative pictures only ($p = .244$), neutral and negative pictures at CPz ($p = .127$), neutral pictures only at CPz ($p = .439$), and negative pictures only at CPz ($p = .062$). Moreover, we did not find a significant relationship between expectations of efficacy regarding the nasal spray and average sustained LPP activity when activity for neutral and negative pictures was collapsed ($p = .834$), neural pictures only ($p = .970$), negative pictures only ($p = .756$), neutral and negative pictures at CPz ($p = .057$), neutral pictures only at CPz ($p = .135$), and negative pictures only at CPz ($p = .075$). Although some associations were marginally significant, we are not confident in the robustness of these relationships. We elaborate on this lack of robust relationship between general beliefs in the efficacy of non-deceptive placebos and expectations of efficacy regarding the nasal spray with sustained LPP activity in the Discussion section of the main manuscript.

As expected, however, the different ways to measure the sustained LPP are all significantly correlated with each other (all p values < .001).

Supplementary Table 6: Early LPP contrasts for Condition by Time by Anterior/Posterior interaction

		Control	Non-deceptive placebo			
**Time Window**	**Anterior vs. Posterior**	***M (SEM)***	***M (SEM)***	***t***	***p***	***d***
400–700 ms	Anterior	-4.27 (.48)	-5.49 (.48)	1.79	.075	.25
400–700 ms	Posterior	3.66 (.39)	3.40 (.39)	.47	.636	.07
700–1000 ms	Anterior	1.49 (.46)	.49 (.46)	1.56	.120	.22
700–1000 ms	Posterior	3.22 (.38)	2.52 (.38)	1.30	.194	.18

Note. This table represents detailed statistics for multiple comparisons for a significant Condition by Time by Anterior/Posterior three-way interaction (p = .047) with two samples combined (total participants, n = 198; control group, n = 99; non-deceptive placebo group, n = 99). The table is broken down by Time, then Anterior/Posterior sites. All follow-up independent pairwise comparisons were two-tailed with unadjusted p values. The comparison was control minus non-deceptive placebo. Positive t and d indicate beneficial effects of non-deceptive placebos.

Supplementary Table 7: Early LPP contrasts for Condition by Time by Picture Type by Hemisphere by Anterior/Posterior interaction

				Control	Non-deceptive placebo			
**Time Window**	**Picture Type**	**Hemisphere**	**Anterior vs. Posterior**	***M (SEM)***	***M (SEM)***	***t***	***p***	***d***
400–700ms	Neutral	Left	Anterior	-5.14 (.48)	-6.28 (.48)	1.67	.095	.24
400–700 ms	Neutral	Left	Posterior	.62 (.44)	.06 (.44)	.91	.365	.13
400–700ms	Neutral	Right	Anterior	-4.23 (.47)	-5.49 (.47)	1.88	.061	.27
400–700 ms	Neutral	Right	Posterior	2.28 (.43)	1.90 (.43)	.62	.535	.09
400–700ms	Negative	Left	Anterior	-4.19 (.60)	-5.35 (.60)	1.37	.172	.19
400–700 ms	Negative	Left	Posterior	5.45 (.49)	5.28 (.49)	.25	.806	.03
400–700ms	Negative	Right	Anterior	-3.51 (.59)	-4.84 (.59)	1.58	.116	.22
400–700 ms	Negative	Right	Posterior	6.31 (.46)	6.37 (.46)	-.08	.932	-.01
700–1000 ms	Neutral	Left	Anterior	.51 (.49)	-.57 (.49)	1.56	.119	.22
700–1000 ms	Neutral	Left	Posterior	.70 (.45)	-.36 (.45)	1.68	.095	.24
700–1000 ms	Neutral	Right	Anterior	.98 (.47)	.02 (.47)	1.45	.149	.21
700–1000 ms	Neutral	Right	Posterior	.99 (.44)	.17 (.44)	1.32	.187	.19
700–1000 ms	Negative	Left	Anterior	2.05 (.57)	1.14 (.57)	1.13	.261	.16
700–1000 ms	Negative	Left	Posterior	5.71 (.47)	5.03 (.47)	1.02	.309	.14
700–1000 ms	Negative	Right	Anterior	2.43 (.55)	1.35 (.55)	1.41	.161	.20
700–1000 ms	Negative	Right	Posterior	5.47 (.46)	5.24 (.46)	.36	.715	.05

Note. This table represents detailed statistics for multiple comparisons for a significant Condition by Picture Type by Time by Hemisphere by Anterior/Posterior five-way interaction with two samples combined (total participants, n = 198; control group, n = 99; non-deceptive placebo group, n = 99). The table is broken down by Time, Picture Type, Hemisphere, and Anterior/Posterior sites. All follow-up independent pairwise comparisons were two-tailed with unadjusted p values. The comparison was control minus non-deceptive placebo. Positive t and d indicate beneficial effects of non-deceptive placebos.

None of the contrasts from these interactions revealed any consistent Condition effect. Therefore, we are not confident in any effect of non-deceptive placebos on the early LPP.

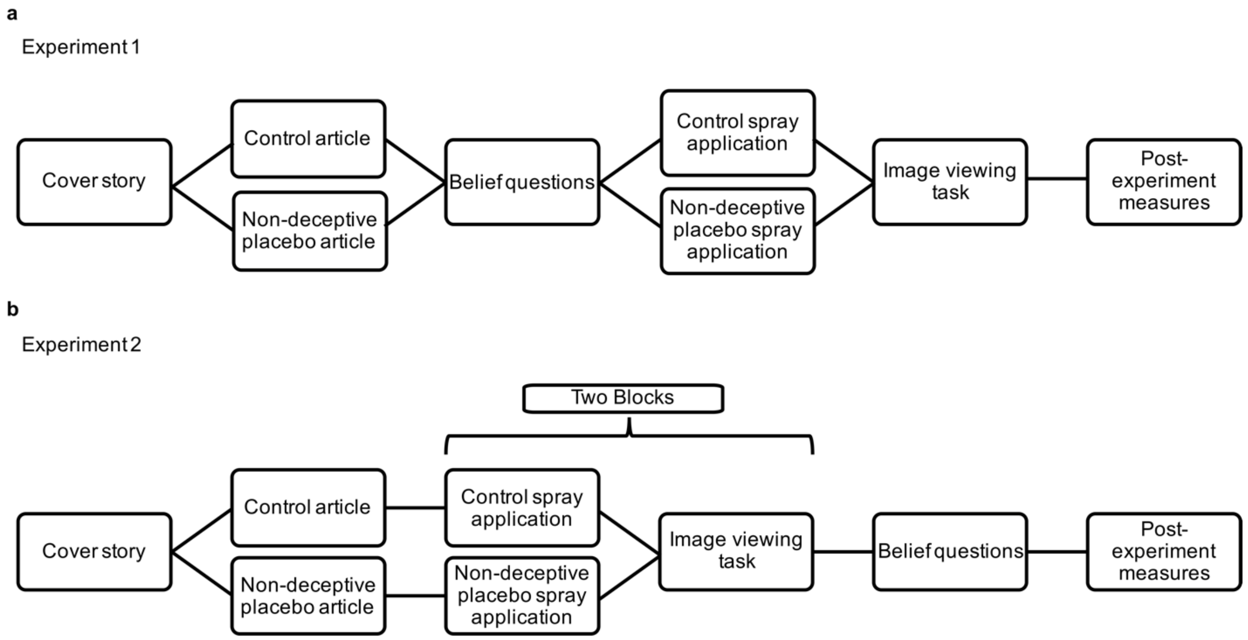

Supplementary Figure 3: Experimental design diagram. This diagram depicts the design of both experiments across session time. Single blocks represent that participants experienced similar instructions. Double blocks represent participants receiving different instructions. Detailed information regarding each block can be found in the Methods section of the main manuscript. **a** Depicts Experimental 1 design. Article information can be found in Supplementary Methods 1. Belief and post-experiment measures can be found in Supplementary Methods 3. The description of images can be found in Supplementary Table 10. **b** Depicts Experimental 2 design. Article information and added manipulation instructions can be found in Supplementary Methods 1 and Supplementary Methods 2. Belief and post-experiment measures can be found in Supplementary Methods 4. The description of images can be found in Supplementary Table 11.

Supplementary Table 8: Preliminary analysis and results for Experiment 1

	**Control**	**Non-deceptive placebo**			
**Variables**	***M (SEM)***	***M (SEM)***	***t***	***p***	***d***
Duration of reading time (secs)	510.01 (29.98)	496.31 (33.52)	.31	.761	.08
Perception of article quality	6.62 (.26)	7.15 (.23)	-1.53	.132	-.39
Beliefs in non-deceptive placebos	3.06 (.29)	4.64 (.38)	-3.34	.001	-.85
Post-article reading mood	6.16 (.23)	6.41 (.26)	-.75	.455	-.19
Effectiveness of nasal spray	1.30 (.36)	2.24 (.44)	-1.66	.101	-.42

Note. A total of $n = 62$ participants was submitted for analyses for all variables except post-article reading mood ($n = 61$) because one participant did not provide a response; Control group, $n = 33$ (except for post-article reading mood, $n = 32$) and Non-deceptive placebo, $n = 29$. We conducted separate independent-samples t tests for each variable (two-tailed; unadjusted). The comparison was control minus non-deceptive placebo. Positive t and d indicate lower non-deceptive placebo values.

Participants did not differ in reading duration and perception of article quality (p values > .05). We tested whether our article manipulation was successful in changing people's beliefs about the effectiveness of non-deceptive placebos. As the control group suggest, most people do not believe in the effectiveness of placebos without deception ($M = 3.06$, $SD = 1.69$) compared to the midpoint (5 on a 0 to 10 Likert scale), $t(32) = -6.58$, $p < .001$, $-d = 1.14$. However, those in the non-deceptive placebo group ($M = 4.64$, $SD = 2.03$) significantly believed in the effectiveness of non-deceptive placebos compared to the control group, $t(60) = -3.34$, $p = .001$, $d = -.85$. It is important to point out that although that our placebo article changed people's beliefs regarding the effectiveness of non-deceptive placebos compared to the control group, our placebo article did change it above the midpoint, suggesting there is difficulty in convincing people of their beliefs regarding non-deceptive placebos. Moreover, we did not detect a significant difference in mood after reading the article, suggesting that the control article was not a negative mood induction ($p = .455$). Lastly, although trending in the hypothesized direction, participants in the non-deceptive placebo group did not perceive the nasal spray as more effective compared to those in the control group ($p = .101$).

Supplementary Table 9: Preliminary analysis and results for Experiment 2

	**Control**	**Non-deceptive Placebo**			
**Variables**	***M (SEM)***	***M (SEM)***	***t***	***p***	***d***
Duration of reading time (secs)	828.52 (21.76)	798.86 (23.46)	.93	.355	.13
Duration of writing time (secs)	265.74 (16.88)	299.46 (13.63)	-1.56	.122	-.22
Perception of article quality	7.69 (.11)	7.62 (.12)	.48	.630	.07
Beliefs in non-deceptive placebos	3.81 (.23)	8.11 (.21)	-13.84	< .001	-1.97
Effectiveness of nasal spray	1.23 (.05)	1.64 (.07)	-4.81	< .001	-.68
Perception of experimenter warmth	6.09 (.11)	5.95 (.13)	.89	.376	.13
Perception of experimenter competence	6.53 (.05)	6.43 (.08)	1.00	.316	.14
Optimism	15.09 (.36)	14.25 (.42)	1.53	.129	.22
Tendency to Worry	55.38 (1.24)	55.82 (1.45)	-.23	.820	-.03
Trait Anxiety	39.87 (.95)	42.34 (.99)	-1.80	.073	-.26
Social Desirability Responding	17.27 (.51)	16.40 (.43)	1.30	.197	.18

Note. Combining two samples, a total of n = 198 participants was submitted for analyses with n = 99 in the control group, and n = 99 in the non-deceptive placebo group. We conducted separate independent-samples t tests for each variable (two-tailed; unadjusted). The comparison was control minus non-deceptive placebo. Positive t and d indicate lower non-deceptive placebo values.

No significant differences emerge for the duration of reading and writing and perception of quality of the article readings (p values > .05). These items were assessed during the article reading phase of the study (see Supplementary Figure 3b for details). It is important to point out that the next set of measures were assessed at the end of the study. There were no significant differences in perception of experimenter warmth and competence, optimism, tendency to worry, and tendency to social desirably respond (all p values > .05). However, it appears that those in the non-deceptive group reported higher trend levels of trait anxiety compared to the control group. When we controlled for trait anxiety in our overall model for the sustained LPP, it was not a significant predictor (p = .221). More importantly, it did not significantly alter the main effect of Condition, $F(1, 193) = 9.80$, $p = .002$, $\eta_p^2 = .048$, and the Condition by Time interaction, $F(1.62, 312.62) = 4.81$, $p = .014$, $\eta_p^2 = .024$. We also controlled for the perception of experimenter warmth and competence and found that it did not significantly impact our results.

Importantly, as a manipulation check, our non-deceptive placebo manipulation was effective in changing people's beliefs about non-deceptive placebos and their expectations of the nasal spray working (p < .001).

Supplementary Table 10: IAPS images for Experiment 1

Picture Type	Number	Picture Type	Number
Neutral	6150	Negative	3130
Neutral	7006	Negative	3140
Neutral	7009	Negative	3150
Neutral	7035	Negative	3170
Neutral	7080	Negative	3180
Neutral	7090	Negative	3350
Neutral	7100	Negative	3400
Neutral	7150	Negative	3550
Neutral	7190	Negative	6230
Neutral	7233	Negative	6350
Negative	3010	Negative	6360
Negative	3030	Negative	6510
Negative	3051	Negative	6550
Negative	3053	Negative	6560
Negative	3060	Negative	6570
Negative	3071	Negative	9040
Negative	3080	Negative	9250
Negative	3100	Negative	9410
Negative	3110	Negative	9420
Negative	3120	Negative	9921

Note. IAPS images for Experiment 1 presented for one block.

Supplementary Note 3: Regarding skin conductance data for Experiment 1

We initially intended to collect skin conductance response data. Unfortunately, due to frequent equipment malfunction, we were unable to record this data reliably. We continued with the study since the primary focus was on self-report ratings of emotional distress. We still used the equipment as part of the study since it played an essential role in our cover story regarding the nasal spray improving physiological readings.

Supplementary Table 11: IAPS images for Experiment 2

Picture Type	Number	Picture Type	Number	Picture Type	Number
Neutral	2190	Neutral	2200	Neutral	2210
Neutral	2230	Neutral	2570	Neutral	2840
Neutral	5500	Neutral	5531	Neutral	7000
Neutral	7002	Neutral	7009	Neutral	7010
Neutral	7020	Neutral	7025	Neutral	7035
Neutral	7050	Neutral	7080	Neutral	7100
Neutral	7150	Neutral	7160	Neutral	7170
Neutral	7175	Neutral	7190	Neutral	7217
Neutral	7224	Neutral	7233	Neutral	7235
Neutral	7550	Neutral	7700	Neutral	5950
Negative	2688	Negative	6312	Negative	6313
Negative	6825	Negative	9425	Negative	9428
Negative	9620	Negative	9622	Negative	9908
Negative	3181	Negative	3350	Negative	3500
Negative	3530	Negative	6212	Negative	6821
Negative	2683	Negative	2811	Negative	3301
Negative	6550	Negative	6520	Negative	8485
Negative	9050	Negative	9183	Negative	9414
Negative	6242	Negative	6231	Negative	6230
Negative	3170	Negative	3220	Negative	9903

Note. IAPS images for Experiment 2 were presented in two blocks.

Supplementary Table 12: Bootstrap method on the sustained LPP

	Control	Non-deceptive placebo			
**Sustained LPP (1000–6000 ms)**	***M (SEM)***	***M (SEM)***	***t***	***p***	***d***
Cluster images collapsed	2.86 (.44)	.85 (.49)	3.00	.004	.43
Cluster neutral images	1.55 (.59)	-.59 (.57)	2.57	.010	.37
Cluster negative images	4.17 (.53)	2.30 (.60)	2.33	.022	.33
CPz images collapsed	2.50 (.62)	-.04 (.67)	2.73	.006	.40
CPz neutral images	.57 (.81)	-1.91 (.73)	2.26	.024	.32
CPz negative images	4.42 (.67)	1.83 (.81)	2.45	.014	.35

Note. Combining two samples, a total of *n* = 198 participants was submitted for analyses with *n* = 99 in the control group, and *n* = 99 in the non-deceptive placebo group. We conducted bootstrap independent-samples *t* tests for each variable (10000 iterations; two-tailed; no adjustments for multiple comparisons). The comparison was control minus non-deceptive placebo. Cluster mean amplitudes were calculated from the mean activity of all respective sites (see Methods for details) from 1000 to 6000 ms. Mean CPz amplitudes were calculated from 1000 to 6000 ms. SEM reflects bias-corrected SEM. Positive *t* and *d* indicate beneficial effects of non-deceptive placebos.

We first calculated several composite sustained LPP (1000–6000 ms) variables at the cluster and CPz level. We performed several procedures to detect outliers, including visual inspection of boxplots, Cook’s distance calculations, and standard deviation calculations. Although we do not detect extreme outliers with boxplots and Cook’s distance calculations, four participants were 3± standard deviations from the mean at the cluster level and two participants at the CPz level. To test the robustness of our main effect findings, we performed bootstrap independent-samples *t* tests. We found identical effects not only with activity from each Picture Type collapsed but also separately for neutral and negative images. Therefore, our main effect findings are robust against these potential extreme values.

Supplementary Methods 3: Additional measures for Experiment 1

To test that our placebos without deception manipulation did not differ in other important elements from the control group, we measured key variables such as duration of reading time and quality of the article readings. As a manipulation check, if our article readings did what it was intended to do, we measured participant's beliefs in the effectiveness of placebos without deception; moreover, we measured participant's perception of the overall effectiveness of the nasal spray. For exploratory purposes, we measured trait-type constructs such as dispositional optimism[33], need for cognition[34], and Big-Five personality traits[35] at the end of the study; in addition, we obtained open-ended data on what participant's thought regarding different elements of the study[k].

Duration of reading time. The article readings were divided into twelve distinct web pages, each with its own time counter[l]. The duration of the reading time was estimated as the total amount of time in seconds a participant stayed on each web page that contained the article information (M = 503.60 secs, SD = 174.82 secs).

Quality of article readings. To assess the quality of the article readings, participants rated 6-items on the extent the information was Effectively conveyed, Convincing, Novel, Interesting, Well-written, and Useful on an 11-point Likert scale from 0 (Not at all) to 10 (Extremely). The six items were reliable and averaged to form a composite quality score with higher numbers indicating higher quality (α = .83, M = 6.87, SD = 1.39)[m].

Beliefs in the effectiveness of placebos without deception. As a manipulation check that our article reading successfully changed people's beliefs regarding the effectiveness of placebos without deception, participants answered three items on an 11-point scale from 0 (Extremely unlikely) to 10 (Extremely likely): "To what extent do you think a placebo would work on you if you knew you were taking a placebo?", "To what extent do you think you need to be deceived into thinking you are taking a real treatment in order for a placebo to work?" (reverse coded), "To what extent do you think a placebo would reduce your negative emotions if you knew you were taking a placebo?". These items were embedded in other belief items on memories and placebos. These items were reliable and averaged to form a composite score on beliefs in the effectiveness of placebos without deception (α = .74, M = 3.80, SD = 2.01). Higher numbers indicate greater belief placebos without deception can work.

Effectiveness of nasal spray. To explore whether people believed that the nasal spray was effective in reducing their negative emotions, we asked participants, on an 11-point Likert scale with 0 (Not at all effective) and 10 (Very Effective), "How effective do you think the nasal spray was in helping you reduce your negative emotions?" Higher numbers indicate greater perception of effectiveness (M = 1.74, SD = 2.25). It is important to note that those in the control group did not receive any information regarding any regulating effects of the nasal spray or any explanation of why there were being asked this question. Thus, it may seem odd to those participants to be asked this question, so interpretation of the data should keep this in mind.

---

[k] We do not report any findings from these exploratory variables here.

[l] Unfortunately, we are unable to use the duration on the first web page because in some cases, the experimenter made an error in having the web page up with the timer counting before the participant began reading the information. Thus, the composite score of the reading time were from the rest of the eleven web pages.

[m] Two participants had missing value and were omitted for reliability analysis.

Supplementary Methods 4: Additional measures for Experiment 2

Similar to Experiment 1, we measured additional variables such as duration of reading time, duration of writing time, quality of the article manipulations, beliefs in the effectiveness of placebos without deception, the effectiveness of the nasal spray, and perception of the experimenters. For exploratory purposes, we also measured dispositional optimism[33], tendency to worry[36], trait anxiety[37], Big-Five personality traits[35], symptoms of depression and anxiety[38], and social desirability[39] at the end of the study; in addition, we obtained open-ended data on what participant's thought of regarding different elements of the study[n].

Duration of reading and writing time. The article readings were divided into eleven distinct web pages similar to Experiment 1. To ensure that participants adequately read the material, we set a minimum timer for each web page ranging from 10 to 60 seconds. The duration of reading time was estimated as the total amount of time in seconds a participant stayed on each web page (M = 842.27 secs, SD = 246.14 secs). We also asked participants to write what they learned and recorded the amount of time they wrote to use as a proxy for engagement with the reading material (M = 286.04 secs, SD = 173.67 secs).

Quality of article manipulation. To assess the quality of the article manipulations, participants rated twelve items on the extent the information was Effectively conveyed, Convincing, Novel, Interesting, Well-written, Useful, Difficult to understand, Credible, Persuasive, Believable, Doubtful, and Unrealistic on an 11-point Likert scale from 0 (Not at all) to 10 (Extremely). Difficult to understand, Doubtful, and Unrealistic were reverse coded. The twelve items were reliable and averaged to form a composite score of article quality with higher numbers indicating greater quality (α = .83, M = 7.55, SD = 1.19)[o].

Beliefs in the effectiveness of placebos without deception. As a manipulation check that our article reading successfully changed people's expectations regarding the effectiveness of placebos without deception, participants rated five statements on an 11-point scale from 0 (Definitely not true) to 10 (Definitely true): "A placebo can still work on me even though I know that I am taking a placebo," "In order for placebos to work, the person needs to be deceived into believing they are taking an actual medicine," "A placebo can reduce my negative emotions even though I know I am taking a placebo," "A placebo only works if the person is deceived into thinking they are taking an actual medicine," and "A placebo can reduce my pain even though I know that I am taking a placebo." These items were embedded in other belief items on pain. Three of the statements were reverse coded; these items were reliable and averaged to form a composite score on beliefs in the effectiveness of placebos without deception (α = .94, M = 5.79, SD = 3.07). Higher numbers indicate a greater belief in the effectiveness of placebos without deception.

Effectiveness of nasal spray. To explore whether people believed that the nasal spray was effective in reducing their negative emotions, we asked participants in the placebos without deception group, on a 3-point scale with 1 as No, 2 as Maybe, and 3 as Yes, "Do you think the placebo nasal spray reduced your negative emotional response to the pictures?" For the control group, we did not want to ask the question out of the blue, so we prefaced the question and asked, "Sometimes, the saline nasal spray has an incidental effect of reducing people's negative emotional response. Do you think the saline nasal spray reduced your negative emotional response to the pictures?" It is important to note that those in the control group did not receive

[n] We report some of these findings.
[o] Two participants had missing values and were omitted for reliability analysis.

any information regarding any regulating effects of the nasal spray. Higher numbers indicate greater perception of effectiveness (*M* = 1.39, *SD* = .57).

Perception of experimenters. There is some evidence that the perception of warmth and competence of the experimenter may influence the placebo effect[40]. Therefore, we measured the participant's perception of our experimenters. Each experimental session was conducted with two experimenters with one of them designated as the primary experimenter. The primary experimenter communicated the most with the participant, provided the verbal suggestion manipulation, and administered the nasal spray. The secondary experimenter assisted the primary experimenter and minimally interacted with the participant. To measure competence and warmth, participants rated each experimenter on the extent they were Competent, Knew what he/she was doing, Authority, Easy to understand, Confident, Likeable, Warm, and Cold on a 7-point Likert scale from 1 (Not at all) to 7 (Extremely). The item Cold was reverse coded. A composite score was averaged for warmth (Likeable, Warm, Cold; $\alpha = .87$, *M* = 5.60, *SD* = 1.34) and competence (Competent, Knew what he/she was doing, Authority, Easy to understand, Confident; $\alpha = .90$, *M* = 6.37, *SD* = .82) for the primary experimenter[p]. A higher number indicates a greater perception of warmth and competence.

[p] We only include information on the primary experimenter since he interacted the most with the participant.

References

1. Colloca L, Howick J. Placebos without deception: A review of their outcomes, mechanisms, and ethics. *International Review of Neurobiology* **138**, 219-240 (2018).

2. Kaptchuk TJ*, et al.* Placebos without deception: A randomized controlled trial in irritable bowel syndrome. *PLOS ONE* **5**, e15591 (2010).

3. Park LC, Covi L. Nonblind placebo trial: an exploration of neurotic patients' responses to placebo when its inert content is disclosed. *Archives of General Psychiatry* **12**, 336-345 (1965).

4. Sandler AD, Bodfish JW. Open-label use of placebos in the treatment of ADHD: A pilot study. *Child: Care, Health and Development* **34**, 104-110 (2008).

5. Sandler AD, Glesne CE, Bodfish JW. Conditioned placebo dose reduction: A new treatment in ADHD? *Journal of Developmental Behavioral Pediatrics* **31**, 369-375 (2010).

6. Kelley JM, Kaptchuk TJ, Cusin C, Lipkin S, Fava M. Open-label placebo for major depressive disorder: A pilot randomized controlled trial. *Psychotherapy and Psychosomatics* **81**, 312-314 (2012).

7. Kam-Hansen S*, et al.* Altered placebo and drug labeling changes the outcome of episodic migraine attacks. *Science Translational Medicine* **6**, 218ra215 (2014).

8. Carvalho C, Caetano JM, Cunha L, Rebouta P, Kaptchuk TJ, Kirsch I. Open-label placebo treatment in chronic low back pain: A randomized controlled trial. *Pain* **157**, 2766–2772 (2016).

9. Schaefer M, Harke R, Denke C. Open-label placebos improve symptoms in allergic rhinitis: A randomized controlled trial. *Psychotherapy and Psychosomatics* **85**, 373-374 (2016).

10. Mundt JM, Roditi D, Robinson ME. A comparison of deceptive and non-deceptive placebo analgesia: Efficacy and ethical consequences. *Annals of Behavioral Medicine* **51**, 307-315 (2016).

11. Locher C, Nascimento AF, Kirsch I, Kossowsky J, Meyer A, Gaab J. Is the rationale more important than deception? A randomized controlled trial of open-label placebo analgesia. *Pain* **158**, 2320-2328 (2017).

12. Meeuwis SH*, et al.* Placebo effects of open-label verbal suggestions on itch. *Acta Dermato-Venereologica* **98**, 268-274 (2017).

13. Schaefer M, Sahin T, Berstecher B. Why do open-label placebos work? A randomized controlled trial of an open-label placebo induction with and without extended information about the placebo effect in allergic rhinitis. *PLOS ONE* **13**, e0192758 (2018).

14. Hoenemeyer TW, Kaptchuk TJ, Mehta TS, Fontaine KR. Open-label placebo treatment for cancer-related fatigue: A randomized-controlled clinical trial. *Scientific Reports* **8**, 2784 (2018).

15. Zhou ES, Hall KT, Michaud AL, Blackmon JE, Partridge AH, Recklitis CJ. Open-label placebo reduces fatigue in cancer survivors: A randomized trial. *Supportive Care in Cancer* **27**, 2179–2187 (2018).

16. Mathur A, Jarrett P, Broadbent E, Petrie KJ. Open-label placebos for wound healing: A randomized controlled trial. *Annals of Behavioral Medicine* **52**, 902-908 (2018).

17. El Brihi J, Horne R, Faasse K. Prescribing placebos: An experimental examination of the role of dose, expectancies, and adherence in open-label placebo effects. *Annals of Behavioral Medicine* **53**, 16-28 (2019).

18. Leibowitz KA, Hardebeck EJ, Goyer JP, Crum AJ. The role of patient beliefs in open-label placebo effects. *Health Psychology* **38**, 613-622 (2019).

19. Barnes K, Yu A, Josupeit J, Colagiuri B. Deceptive but not open label placebos attenuate motion-induced nausea. *Journal of Psychosomatic Research* **125**, 109808 (2019).

20. Schaefer M, Denke C, Harke R, Olk N, Erkovan M, Enge S. Open-label placebos reduce test anxiety and improve self-management skills: A randomized-controlled trial. *Scientific Reports* **9**, 13317 (2019).

21. Swafford AP, Kwon DP, MacLennan RJ, Fukuda DH, Stout JR, Stock MS. No acute effects of placebo or open-label placebo treatments on strength, voluntary activation, and neuromuscular fatigue. *European Journal of Applied Physiology* **119**, 2327–2338 (2019).

22. Saunders B*, et al.* "I put it in my head that the supplement would help me": Open-placebo improves exercise performance in female cyclists. *PLOS ONE* **14**, e0222982 (2019).

23. Bernstein MH*, et al.* Are conditioned open placebos feasible as an adjunctive treatment to opioids? Results from a single-group dose-extender pilot study with acute pain patients. *Psychotherapy and Psychosomatics* **88**, 380-392 (2019).

24. Meeuwis SH*, et al.* Antipruritic placebo effects by conditioning H1-antihistamine. *Psychosomatic Medicine* **81**, 841-850 (2019).

25. Kleine-Borgmann J, Schmidt K, Hellmann A, Bingel U. Effects of open-label placebo on pain, functional disability, and spine mobility in patients with chronic back pain: A randomized controlled trial. *Pain* **160**, 2891-2897 (2019).

26. Rossow LM, Moon J, Hiebert L, Espitia C, Fahs CA. The effects of honest and dishonest placebo ingestion immediately prior to VO2peak and handgrip strength testing. *Journal of Trainology* **8**, 27-30 (2019).

27. Kube T*, et al.* Deceptive and non-deceptive placebos to reduce pain-An experimental study in healthy people. *The Clinical Journal of Pain* **36**, 68-79 (2020).

28. Geers AL, Briñol P, Vogel EA, Aspiras O, Caplandies FC, Petty RE. The application of persuasion theory to placebo effects. *International Review of Neurobiology* **138**, 113-136 (2018).

29. Wager TD*, et al.* Placebo-induced changes in fMRI in the anticipation and experience of pain. *Science* **303**, 1162-1167 (2004).

30. Kirsch I, Deacon BJ, Huedo-Medina TB, Scoboria A, Moore TJ, Johnson BT. Initial severity and antidepressant benefits: A meta-analysis of data submitted to the Food and Drug Administration. *PLOS Medicine* **5**, e45 (2008).

31. Petrovic P, Dietrich T, Fransson P, Andersson J, Carlsson K, Ingvar M. Placebo in emotional processing—induced expectations of anxiety relief activate a generalized modulatory network. *Neuron* **46**, 957-969 (2005).

32. Goetz CG, Leurgans S, Raman R, Stebbins GT. Objective changes in motor function during placebo treatment in PD. *Neurology* **54**, 710-710 (2000).

33. Scheier MF, Carver CS, Bridges MW. Distinguishing optimism from neuroticism (and trait anxiety, self-mastery, and self-esteem): A reevaluation of the life orientation test. *Journal of Personality and Social Psychology* **67**, 1063-1078 (1994).

34. Cacioppo JT, Petty RE, Feng Kao C. The efficient assessment of need for cognition. *Journal of Personality Assessment* **48**, 306-307 (1984).

35. Gosling SD, Rentfrow PJ, Swann WB, Jr. A very brief measure of the Big-Five personality domains. *Journal of Research in Personality* **37**, 504-528 (2003).

36. Meyer TJ, Miller ML, Metzger RL, Borkovec TD. Development and validation of the Penn State Worry Questionnaire. *Behaviour Research and Therapy* **28**, 487-495 (1990).

37. Spielberger CD, Gorsuch RL, Lushene RE. *Manual for the state-trait anxiety inventory*. Consulting Psychologists Press (1970).

38. Watson D, Clark LA, Weber K, Assenheimer JS, Strauss ME, McCormick RA. Testing a tripartite model: II. Exploring the symptom structure of anxiety and depression in student, adult, and patient samples. *Journal of Abnormal Psychology* **104**, 15-25 (1995).

39. Crowne DP, Marlowe D. A new scale of social desirability independent of psychopathology. *Journal of Consulting Psychology* **24**, 349-354 (1960).

40. Howe LC, Goyer JP, Crum AJ. Harnessing the placebo effect: Exploring the influence of physician characteristics on placebo response. *Health Psychology* **36**, 1074-1082 (2017).
