# Supplementary material for: Placebos without deception reduce self-report and neural measures of emotional distress
Source: Nat Commun. 2020 Jul 29;11:3785. doi: 10.1038/s41467-020-17654-y (PMC7391658; doi:10.1038/s41467-020-17654-y)
Supplement: Supplementary file 3 — Reporting Summary [file 41467_2020_17654_MOESM3_ESM.pdf]

nature research

Corresponding author(s): Darwin A. Guevarra

Last updated by author(s): June 13, 2020

Reporting Summary

Nature Research wishes to improve the reproducibility of the work that we publish. This form provides structure for consistency and transparency in reporting. For further information on Nature Research policies, see Authors & Referees and the Editorial Policy Checklist .

Statistics

For all statistical analyses, confirm that the following items are present in the figure legend, table legend, main text, or Methods section.

n/a	Confirmed	
☐	☒	The exact sample size (*n*) for each experimental group/condition, given as a discrete number and unit of measurement
☐	☒	A statement on whether measurements were taken from distinct samples or whether the same sample was measured repeatedly
☐	☒	The statistical test(s) used AND whether they are one- or two-sided<br>*Only common tests should be described solely by name; describe more complex techniques in the Methods section.*
☐	☒	A description of all covariates tested
☐	☒	A description of any assumptions or corrections, such as tests of normality and adjustment for multiple comparisons
☐	☒	A full description of the statistical parameters including central tendency (e.g. means) or other basic estimates (e.g. regression coefficient) AND variation (e.g. standard deviation) or associated estimates of uncertainty (e.g. confidence intervals)
☐	☒	For null hypothesis testing, the test statistic (e.g. *F*, *t*, *r*) with confidence intervals, effect sizes, degrees of freedom and *P* value noted<br>*Give P values as exact values whenever suitable.*
☒	☐	For Bayesian analysis, information on the choice of priors and Markov chain Monte Carlo settings
☐	☒	For hierarchical and complex designs, identification of the appropriate level for tests and full reporting of outcomes
☐	☒	Estimates of effect sizes (e.g. Cohen's *d*, Pearson's *r*), indicating how they were calculated

Our web collection on statistics for biologists contains articles on many of the points above.

Software and code

Policy information about availability of computer code

Data collection	All picture stimuli was presented with E-Prime (version 2.0) presentation software. All EEG data were collected with a 64 channel Active Two Biosemi System.
Data analysis	Data analysis was conducted through SPSS version 26. EEG data was processed using BrainVision Analyzer 2.2. Figure 1b was created using R Studio (version 3.6.1) and ggplot2 (version 3.3.0). Figures 2b and Figures 2d, Supplementary Figure 1a and 1b, and Supplementary Figure 2a and 2b were created with SigmaPlot (version 14). Topographic headmaps for Figure 2c was created with BrainVision Analyzer 2.2.

For manuscripts utilizing custom algorithms or software that are central to the research but not yet described in published literature, software must be made available to editors/reviewers. We strongly encourage code deposition in a community repository (e.g. GitHub). See the Nature Research guidelines for submitting code & software for further information.

Data

Policy information about availability of data

All manuscripts must include a data availability statement. This statement should provide the following information, where applicable:

- Accession codes, unique identifiers, or web links for publicly available datasets
- A list of figures that have associated raw data
- A description of any restrictions on data availability

Data supporting these findings can be found at the Open Science Framework (https://osf.io/s3b8d/). SPSS (version 26) is used for all statistical analyses. Data and R Code underlying Fig. 1b can be found in Experiment 1 Data Files. Data and SPSS syntax underlying Fig. 2d, Supplementary Fig. 1b, and Supplementary Figs. 2a and 2b can be found in Experiment 2 Data Files. A reporting summary for this Article is available as a Supplementary Information file. Additional data from these studies are available from the corresponding author upon request.

October 2018

Field-specific reporting

Please select the one below that is the best fit for your research. If you are not sure, read the appropriate sections before making your selection.

☐ Life sciences ☒ Behavioural & social sciences ☐ Ecological, evolutionary & environmental sciences

For a reference copy of the document with all sections, see nature.com/documents/nr-reporting-summary-flat.pdf

Behavioural & social sciences study design

All studies must disclose on these points even when the disclosure is negative.

Study description

We ran two Experiments testing the effectiveness of non-deceptive placebos on emotional distress. Experiment 1 tested non-deceptive placebos on self-report measures of emotional distress. Experiment 2 tested non-deceptive placebos on a neural marker of emotional distress. All data reported are quantitative.

Research sample

For Experiment 1, sixty-eight participants were recruited from a nonclinical sample at a large university in the Midwest. They were compensated with course credit for their time. The final sample submitted to analyses included 62 participants with n = 33 (Mage = 18.61, SD = .83; 39.4% female; 60.6% European American) in the control group and n = 29 (Mage = 18.76, SD = .74; 34.5% female; 75.9% European American) in the non-deceptive placebo. The sample for Experiment 1 is a non-representative sample of the United States population. The sample for this data was a convenience sample to first test whether our non-deceptive placebo manipulation would work on a young adult population.

For Experiment 2, a total of two hundred and eighteen people from a non-clinical sample participated in Experiment 2 from another large university in the Midwest. They were compensated with course credit (n = 110) or $20 (n = 108) for their time. One hundred and ninety-eight participants were submitted to analyses, with n = 99 in the control group (Mage = 19.92, SD = 2.14; 78.8% European American) and n = 99 in the non-deceptive placebo group (Mage = 19.78, SD = 2.36; 80.8% European American). The sample for Experiment 2 is a non-representative sample of the United States population, but is racially a representative sample of East Lansing, Michigan. Moreover, we restricted our sample to female participants to control for well-established sex differences in emotional reactivity (Bradley et al., 2001; Cahill, 2006).

Sampling strategy

For Experiment 1, we used convenience sampling by recruiting from a student sample through the psychology department's participant pool. Students were encouraged to pick different studies to participate as part of a class requirement. For Experiment 2, we used convenience sampling by recruiting from a student sample through the psychology department's participant pool and community sample through flyering and on-line recruitment.

In terms of determining sample size, for Experiment 1, to first test the effectiveness of our manipulation, we simply tried to run as many participants in one semester. For Experiment 2, we initially elected to run 50 participants in each condition (100 total) based on other non-deceptive placebo studies but decided to run 100 in each condition (200 total) to detect a d-effect size of .40 with a power of .8 at p = .05.

Data collection

For both experiments, participants were told that the study was on cognitive processing, memory, and emotion. Participants were randomly assigned to a control or non-deceptive placebo group. Those in the control group read an article on the neurological processes of pain and how to treat it. Those in the non-deceptive placebo group read an article on the placebo effect, how powerful it is for some conditions, and how it can still work even without deception. After reading the articles, the experimenter delivered different pre-nasal-spray instructions to control and non-deceptive placebo participants. For the non-deceptive placebo group, the experimenter summarized the main points of the reading, positively framed that placebos can still work if the participant believes it will, and administered a saline nasal spray once to each nostril. For the control group, the experimenter explained that the saline nasal spray was designed to help obtain better physiological readings. The articles were matched for narrative structure, negatively valence words (control = 62, non-deceptive placebo = 58), and length (control = 1287 words, non-deceptive placebo = 1270 words). Afterwards, participants engaged in an emotion picture viewing task. For Experiment 1, they reported their feelings after each picture. For Experiment 2, their EEG data were collected as they viewed different images.

Participants in the control group were blind to the fact that they were in the control group. Participants in the non-deceptive placebo group were blind to the fact they were participating in a placebo study until right before the administration of the nasal spray. Due to the nature of the manipulation, it was impossible to blind participants in the non-deceptive placebo group. Moreover, the primary experimenter was not blind to the condition since they delivered some part of the verbal manipulation. It is important to note, however, that none of the authors, who were intimately aware of the purpose of the study, ran any participants.

All article reading material were presented with Qualtrics, an on-line survey software. All self-report measures were also obtained through Qualtrics. All images were presented with E-Prime presentation software. For Experiment 1, self-reported affect was obtained through E-Prime (version 2.0). For Experiment 2, all EEG data were collected with a 64 channel Active Two Biosemi System.

Timing

For Experiment 1, data was collected from March 22, 2015 to April 2, 2015. For Experiment 2, Sample 1 was collected from April 25, 2017 to April 27, 2018 and Sample 2 was collected from July 1, 2019 to December 13, 2019.

Data exclusions

Exclusion criteria were pre-established. For Experiment 1, sixty-eight participants participated in the study but six were removed due to experimenter error or substantial deviation from the protocol (n = 3), participant indicating they were a non-native English speaker at exit survey (n = 1), participant indicating that they read the self-report scale incorrectly (n = 1), and software error resulting in no self-reported affective ratings (n = 1).

	For Experiment 2, twenty participants were removed from analysis due to reporting that English was not their native language at the exit survey (n = 1), software error (n = 4), and excessive artifacts due to eye and body movements (n = 15).
Non-participation	No participants in the final sample dropped out or declined to participate in the study.
Randomization	Participants were randomly assigned into the control or non-deceptive placebo group. The main analysis did not include covariates.

Reporting for specific materials, systems and methods

We require information from authors about some types of materials, experimental systems and methods used in many studies. Here, indicate whether each material, system or method listed is relevant to your study. If you are not sure if a list item applies to your research, read the appropriate section before selecting a response.

Materials & experimental systems

n/a	Involved in the study
☒	☐ Antibodies
☒	☐ Eukaryotic cell lines
☒	☐ Palaeontology
☒	☐ Animals and other organisms
☐	☒ Human research participants
☒	☐ Clinical data

Methods

n/a	Involved in the study
☒	☐ ChIP-seq
☒	☐ Flow cytometry
☒	☐ MRI-based neuroimaging

Human research participants

Policy information about studies involving human research participants

Population characteristics	See above.
Recruitment	For Experiment 1, we recruited from a student sample through the psychology department's participant pool. Students were encouraged to pick different studies to participate in as part of a class requirement. For Experiment 2, we recruited from a student sample through the psychology department's participant pool and community samples through flyering and on-line recruitment.<br><br>Previous non-deceptive placebo studies advertised their study as a "novel mind-body" intervention. We tried to minimize selection bias by advertising our study as a study on Cognitive Processing, Memory, and Emotion. Participants who decide to participant in our study are not even aware they are participating in a non-deceptive placebo study. Therefore, we are not that concerned with selection bias that we recruited participants who would be intrigued and willing to participate in an intervention study.
Ethics oversight	Experiment 1 was approved by the University of Michigan’s Institutional Review Board. Experiment 2 was approved by Michigan State University’s Institutional Review Board

Note that full information on the approval of the study protocol must also be provided in the manuscript.
